# BMP and NODAL paracrine signalling regulate the totipotent-like cell state in embryonic stem cells

**DOI:** 10.3389/fcell.2025.1720355

**Published:** 2026-01-23

**Authors:** Sanidhya Jagdish, Loick Joumier, Sabin Dhakal, Gilberto Duran-Bishop, Mohammed Usama, Mohan Malleshaiah

**Affiliations:** 1 Institut de recherches cliniques de Montréal (IRCM), Montreal, QC, Canada; 2 Department of Experimental Medicine, McGill University, Montreal, QC, Canada; 3 Département de biochimie et médecine moléculaire, Université de Montréal, Montreal, QC, Canada

**Keywords:** bone morphonegentic protein (BMP) Signalling, embryonic stem cells, nodal signalling, paracrine signalling, totipotent-like cell state

## Abstract

Cell–cell communication coordinates signalling between cells to guide context-dependent cell fate decisions such as proliferation, differentiation, and lineage specification. Such communication mechanisms are poorly understood in regulating the stem cell states. In this study, we investigate how cell-cell communication regulates cell fate transitions in heterogeneous embryonic stem cell populations, with a particular focus on totipotent-like cells that resemble the two-cell stage embryo. Using single-cell RNA sequencing in combination with computational frameworks, we map ligand–receptor interactions and model downstream regulatory effects across various stem cell states. We functionally validate the predictions by selectively perturbing signalling pathways under specific culture conditions. Our data reveal the key roles of BMP and NODAL (TGF-β) signalling in mediating intercellular communication to shape stem cell identity and heterogeneity. These findings enhance our understanding of the signalling logic that governs early developmental cell fate decisions, providing new insights into stem cell biology with broad implications for regenerative medicine and developmental modelling.

## 1 Introduction

Cell fate determination is fundamental to embryonic development, where a single cell gives rise to all specialized lineages of an organism (Casey et al., 2020). This process is governed by intricate regulatory networks that integrate transcriptional, epigenetic, and environmental signals (Wu and Schmitz, 2023). The accurate coordination of these developmental pathways is essential not only for normal embryonic development but also for maintaining physiological balance (homeostasis) and supporting tissue regeneration in adulthood (Lee et al., 2024). Cells act as dynamic systems, with complex signalling pathways that are closely interconnected through feedback loops and crosstalk (Lee et al., 2024). Systems biology approaches involving single-cell measurements are thus crucial for unravelling the sophisticated mechanisms underlying cell fate decisions (Lee et al., 2024; Haghverdi and Ludwig, 2023).

Beyond intracellular feedback and crosstalk, cell identity and function are controlled by signals from neighbouring cells, creating a dynamic microenvironment (Davies and Albeck, 2018). Such cell–cell communication (CCC) is facilitated by secreted proteins, like growth factors and cytokines, which activate signalling cascades in adjacent cells, leading to changes in their gene expression, epigenetics, metabolism, and functional patterns (Su et al., 2024; Singer, 1992; Wu et al., 2025). CCC occurs in several modes: autocrine (self-regulation), paracrine (localized responses), endocrine (long-range hormonal signalling), and synaptic (rapid neural communication) (Cooper, 2000). At the population level, CCC can coordinate collective behaviours, such as morphogenesis, wound healing, and immune responses (Su et al., 2024), while also producing heterogeneity through signalling gradients (Movasat et al., 2025). Therefore, a detailed understanding of intercellular communication mechanisms is essential for fundamental biology and therapeutic development.

The CCC mechanisms are particularly evident in stem cell niches, where local signalling controls self-renewal, lineage commitment, and plasticity (Morrison and Spradling, 2008; Przybyla and Voldman, 2012). Due to their ability to differentiate into specialized cell types, stem cells provide a suitable model for studying CCC mechanisms in developmental contexts. Pluripotent cells, such as embryonic stem cells (ESCs) and induced pluripotent stem cells (iPSCs), represent the stem cell niche of early embryogenesis (NRC (US) and Research IoMUCotBaBAoSC, 2002; Liang and Zhang, 2013). Their ability to self-renew and differentiate into all embryonic cell types provides a proxy for examining how CCC mechanisms regulate the emergence of lineage-specific cell types (Romito and Cobellis, 2016). The establishment and maintenance of the pluripotent stem cell state itself is regulated by CCC. For instance, fibroblast growth factor (FGF) signalling controls the balance between self-renewal and differentiation of ESCs: activation of the FGF–ERK pathway promotes differentiation, while its inhibition preserves pluripotency (Lanner and Rossant, 2010). Similarly, paracrine signalling through bone morphogenic protein (BMP) also regulates the pluripotent and other heterogeneous cell states of mouse ESCs (Meharwade et al., 2023). ESCs are inherently heterogeneous and often display distinct cell states of early embryogenesis (Meharwade et al., 2023; Singer et al., 2014): for example, totipotent-like, naïve pluripotent, primed, and primitive endoderm (PrEn) cell states. Such heterogeneity impacts the reproducibility of ESC-based models and therapies (Chehelgerdi et al., 2023), but also mirrors the lineage diversity in the early mammalian embryo, making ESCs a physiologically relevant system for studying how distinct cell populations communicate and influence one another (Yang et al., 2021). Thus, ESC heterogeneity provides a unique opportunity to dissect CCC within a controlled, developmentally relevant context.

Of particular interest in the mouse ESC system are totipotent-like cells (TLCs), which display transcriptional signatures reminiscent of the two-cell (2C) stage embryo and can contribute to both embryonic and extraembryonic lineages (Lu and Zhang, 2015). Although rare and transient, their emergence highlights the plasticity of ESC cultures and the influence of extrinsic signals on their identity. Despite the importance of totipotent cells to initiate mammalian embryonic development, the regulatory mechanisms controlling the totipotent cell state are poorly understood. Understanding how TLCs maintain or exit their state is thus key to elucidating early embryo development.

Studying CCC among specific cell states requires high-resolution single-cell measurements. Single-cell RNA sequencing (scRNA-seq) enables detailed profiling of cell states, including their signalling and transcriptional responses (Jovic et al., 2022). Thus, the effective use of scRNA-seq in combination with computational tools can enable the systematic mapping of ligand–receptor interactions, modelling of signalling and transcriptional networks, and prediction of functional outcomes, offering an integrated systems-level approach to understanding how extrinsic signals are processed at the single-cell level in cell fate determination.

In this study, we used scRNA-seq data, coupled with computational frameworks such as CellChat (Jin et al., 2021), NicheNet (Browaeys et al., 2020), and CellOracle (Kamimoto et al., 2023), to infer CCC among cell states of mouse ESCs. We mapped the paracrine signalling landscape of TLCs and identified BMP and NODAL as the key CCC routes to either enhance or diminish the TLC state, respectively. Furthermore, we provide a novel insight into examining BMP and NODAL signalling through a ligand-receptor interaction perspective, revealing how TLCs function as both signal senders and receivers and how the directionality of these interactions influences TLC maintenance and the global dynamics of the ESC system.

## 2 Materials and methods

### 2.1 Single-cell RNA-seq analysis

scRNA-seq data from ESCs cultured in LIF and BMP growth factors (henceforth referred to as LB) and LB with PD0325901, XAV939, Resorcyclic lactone, and SB431542 small molecules (henceforth referred to as LBPXRS) conditions were processed and analyzed in R (v4.5.0) using the same pipeline described in our previous study (Meharwade et al., 2023). All preprocessing, normalization, and statistical thresholds were applied identically to ensure consistency across datasets using the Seurat (v5) (Stuart et al., 2019) library. Matrices were normalized with LogNormalize (scale factor = 10,000) and integrated using ‘IntegrateData’ to account for batch effects. Dimensionality reduction was performed with PCA (30 components) and UMAP. ‘FindNeighbors’ and ‘FindClusters’ (Louvain, resolution = 0.8) were used for clustering. Cell states were annotated based on established marker expression: TLCs (*Gata2*, *Rell1, Amhr2)* (Choi et al., 2017; Macfarlan et al., 2012); pluripotent cells (*Sox2, Nanog, Klf4*) (Malleshaiah et al., 2016; Silva and Smith, 2008; Nichols and Smith, 2009); primed cells (*Zic3*, *T, Fgf8*) (Nichols and Smith, 2009; Yang et al., 2019; Buecker et al., 2014); PrEn cells (*Gata6, Cdh2, Dab2*) (Sc et al., 2014; Artus et al., 2011) (Supplementary Figure S1).

### 2.2 Cell–cell communication analysis

Intercellular communication was investigated using CellChat (Jin et al., 2021), NicheNetR (Browaeys et al., 2020), and CellOracle (Kamimoto et al., 2023), each providing complementary insights into ligand–receptor signalling and gene regulatory dynamics.

#### 2.2.1 CellChat analysis

CellChat (v1.6.1) infers communication networks by modelling interactions between sender and receiver cell populations using a curated database of ligand–receptor pairs (Jin et al., 2021). This framework allows for the identification of dominant signalling pathways and quantification of their contribution to overall intercellular communication. We applied CellChat to quantify interaction networks, determine pathway activity, and compare global signalling patterns between LB and LBPXRS conditions. The description of the code and workflow for CellChat can be found at the GitHub page: https://github.com/sqjin/CellChat/tree/e4f68625b074247d619c2e488d33970cc531e17c/tutorial.

#### 2.2.2 Differential NicheNetR analysis

NicheNetR (v2.2.1) was used to link ligand activity in sender cells with downstream transcriptional responses in receiver cells (Browaeys et al., 2020). For differential analysis, we defined four distinct signalling niches:

1. *Totipotent niche*–totipotent cells as receivers; primed, PrEn, and pluripotent cells as senders.
2. *Pluripotent niche*–pluripotent cells as receivers; primed, PrEn, and totipotent cells as senders.
3. *Primed niche*–primed cells as receivers; totipotent, PrEn, and pluripotent cells as senders.
4. *PrEn niche*–PrEn cells as receivers; primed, pluripotent, and totipotent cells as senders.

Differential ligand–receptor analysis was performed with default parameters to capture significant interactions. The top 250 predicted target genes were retained for prioritization. Ligand–receptor pairs were ranked according to recommended DE-NicheNetR thresholds, and the top 25 ligands per niche were displayed for visualization. The description of the code and workflow for NicheNet can be found at the GitHub page: https://github.com/saeyslab/nichenetr/tree/master/vignettes.

#### 2.2.3 Pathway-focused NicheNetR analysis

We next performed a pathway-specific NicheNetR analysis (Browaeys et al., 2020) focusing on signalling cascades relevant to early development, including BMP, FGF, WNT, TGF-β, and PDGF pathways (Meharwade et al., 2023). The prioritized ligand-receptor pairs were selected from each pathway and merged into one matrix. Ligand–receptor pairs were then filtered and prioritized based on expression levels and predicted activity using an interaction strength threshold of 0.4. Although the default cutoff is 0.25, we increased it to 0.4 to focus on highly probable interactions; raising it further eliminated biologically meaningful interactions that were consistently identified by both CellChat and Differential NicheNet. Ligand-receptor pairs were retained if they were detected in TLC-to/from-cell communication in at least two of the three receiver populations and were exclusive to either the TLC sender or receiver groups. We then generated dot plots to display the expression of selected ligands and receptors across sender and receiver populations in the LB condition.

### 2.3 Validation experiments

#### 2.3.1 Tissue culture and differentiation

Mouse ESCs (R1) were maintained in knockout DMEM supplemented with non-essential amino acids, sodium pyruvate, L-Glutamine, β-mercaptoethanol, 15% FBS (Gibco, #12483020) and Leukemia inhibitory factor (LIF) (1,000 units/mL, Millipore, ESG1107). In LB medium cells were cultured in N2B27 basal media supplemented with 1,000 units/mL LIF (Millipore Sigma, ESG1107) and BMP4 10 ng/mL (Miltenyi Biotech, 130-111-168). Cells were cultured on gelatin-coated plates under standard conditions (37 °C, 5% CO_2_).

To evaluate the contribution of TGFB signalling, cells were treated with 0.8uM LY2109761 (TGFBR1/II inhibitor; MedChem, #700874-71-1) for 48 h. Optimal concentration of the inhibitor was determined using dose-response assay, and downstream pathway activity assessed by Western blot analysis relative to untreated LB controls.

#### 2.3.2 Western blot analysis

Protein lysates were prepared by lysing cells rinsed with PBS using 2X Laemmli buffer (4% SDS, 20% glycerol, 120 mM Tris-HCl pH 6.8). Following a 10 min incubation at 100 °C, the concentration was measured using Nanodrop (Thermofisher). β-mercaptoethanol and bromophenol blue were added to prepare the samples. Proteins were separated on SDS-PAGE gel and transferred to polyvinylidene difluoride (PVDF) membranes. Membranes were blocked in 5% non-fat milk in TBST for 1 h at room temperature and incubated overnight at 4 °C with primary antibodies anti-phospsho-SMAD2 (S465/467)/SMAD3 (S423/425) (Cell Signaling, #8828S; 1:1,000 dilution), anti-SMAD2/3 (Santa Cruz, sc-133098; 1:1,000 dilution), GAPDH (Santa Cruz, sc-32233; 1:5,000 dilution). After washing, membranes were incubated with HRP-conjugated secondary antibodies: anti-mouse (Jackson Immunoresearch, #715035150; 1:10000 dilution), anti-rabbit (#111035045; 1:10000 dilution) for 1 h at room temperature. Membranes were imaged using ImageQuant LAS 4000 (GE Healthcare). Protein bands were visualized using Clarity western ECL substrate (BioRad, #1705061) and quantified using ImageJ. Signal intensity of phospho-proteins was normalized to their respective total protein and GAPDH levels.

#### 2.3.3 Flow cytometry

To determine the proportion of TLCs, cells expressing MERVL-tdTomato were quantified by flow cytometry. Cells were harvested, washed and resuspended with 1X PBS. Flow cytometry was performed on a BD Fortessa machine. Gating was performed sequentially, beginning with exclusion of debris on FSC-A vs. SSC-A, followed by doublet removal using FSC-H vs. FSC-W. The resulting live singlet population was subsequently gated for tdTomato-positive cells. Data were analyzed using FlowJo v10.10.0.

#### 2.3.4 Quantitative PCR (qPCR)

Total RNA was extracted using TRIzol reagent according to the manufacturer’s instructions. Complementary DNA (cDNA) was prepared using the RevertAid H minus Reverse Transcriptase (Thermo Fisher Scientific, EP0451) with 1 μg of total RNA. Reverse transcription was carried out with the standard cycling condition as per the manufacturer’s instructions. cDNA was diluted 30-fold, and quantitative PCR (qPCR) was performed using the PowerUp SYBR Green Master Mix (Thermo Fisher Scientific A25741). Gene expression levels of TLC state markers were normalized to the housekeeping gene (Gapdh), and relative expression was calculated using the ΔΔCt method. The primers used for qPCR are listed below.

**Table:** 

Genes	Primer sequence	
MERVL	F	GAG​GCT​CCA​AAC​AGC​ATC​TCT​A
	R	CTC​TAC​CAC​TTG​GAC​CAT​ATG​AC
Duxf3	F	CAT​GCC​TCA​AAG​AGG​TCC​AT
	R	CTG​GAT​CCA​AGC​TCT​TCC​TG
Zfp352	F	CCC​TGC​AAT​ACA​CAG​CCT​TC
	R	CTC​CTG​TGT​GCT​ACA​GGA​TGC
Tcstv1	F	TTG​CCA​GCC​CAG​AGT​ACA​AG
	R	AGC​AGT​GGC​TTT​GAT​CTT​TGC
Klf4	F	GAA​GGG​AGA​AGA​CAC​TGC​GT
	R	AGT​GGG​GGA​AGT​CGC​TTC​AT
Nanog	F	GTG​TGC​ACT​CAA​GGA​CAG​GT
	R	GCT​CAG​GTT​CAG​AAT​GGA​GGA
Sox2	F	CTC​TGC​ACA​TGA​AGG​AGC​AC
	R	CGG​GAA​GCG​TGT​ACT​TAT​CCT
Gapdh	F	TGT​TCC​TAC​CCC​CAA​TGT​GT
	R	CCT​GCT​TCA​CCA​CCT​TCT​TG

^*^F = forward; R = reverse.

### 2.4 Pseudotime inference

Trajectories were constructed using the Monocle 3 framework (v1.3.7) (Trapnell et al., 2014). The root of the trajectory was defined as the terminal branchpoint nearest to the totipotent-like population, ensuring that the pseudotime variable reflected cell state transitions along natural embryonic development.

### 2.5 In silico gene perturbation

In silico gene perturbation analysis was performed with CellOracle (v0.18.0) (Kamimoto et al., 2023). Cell state-specific Gene Regulatory Networks (GRNs) were first reconstructed after loading CellOracle’s pre-built base GRN using the “load_mouse_scATAC_atlas_base_GRN” function. The pseudotime variable from the Monocle 3 analysis was implemented in the CellOracle object. Perturbed vector fields were generated for transcription factors, cell state markers, or targets of signaling pathways. Knockouts were simulated using the “simulate_shift” function, setting the target gene expression to 0. Grid parameters were set to 40 grid points and a cell density of 0.01 for digitizing the vector field. The description of the code and workflow for CellOracle can be found at the GitHub page: https://morris-lab.github.io/CellOracle.documentation/tutorials/simulation.html.

## 3 Results

### 3.1 Extensive cell–cell communication supports ESC heterogeneity

**FIGURE 1:**
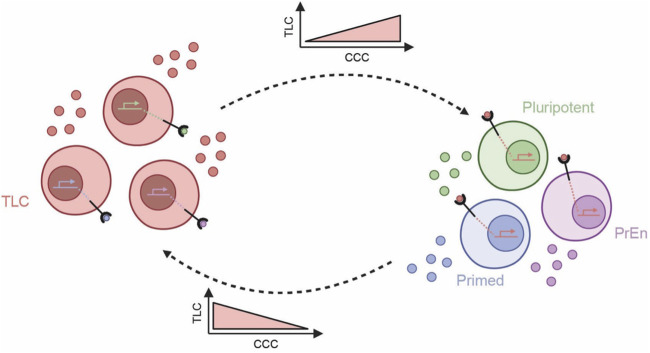
Schematic for the proposed model of signalling interactions regulating the TLC state and its exit. Totipotent-like cells (TLCs) secrete specific ligands that act in a paracrine manner by engaging receptors on neighbouring cell types, thereby transcriptionally reinforcing the TLC state and contributing to a supportive local signalling niche. Increased CCC from TLCs promotes the maintenance and expansion of the TLC state. In contrast, ligands predominantly secreted by pluripotent, primed, or primitive endoderm (PrEn) cells interact with receptors on TLCs to trigger their exit from the TLC state. Increased CCC from the other cell states leads to a reduction in TLCs. Arrows indicate the directionality of signalling interactions.

Intercellular communication is mainly mediated by the interaction of ligands secreted or produced by the “sender” cells with specific receptors on the “receiver” cells. Such ligand-receptor interaction leads to activation of specific cell signalling pathways and their downstream effects in receiver cells. Since the pluripotent cell state in mouse ESCs is sensitive to paracrine signalling effects, we hypothesized that the TLC state is similarly governed by its CCC with other cell states of ESCs (i.e., pluripotent, primed and PrEn) (Figure 1). We envisioned that the extent of the TLC state in the system is regulated by the sender-receiver communication between TLCs and other cell states via the ligand-receptor interactions.

**FIGURE 2:**
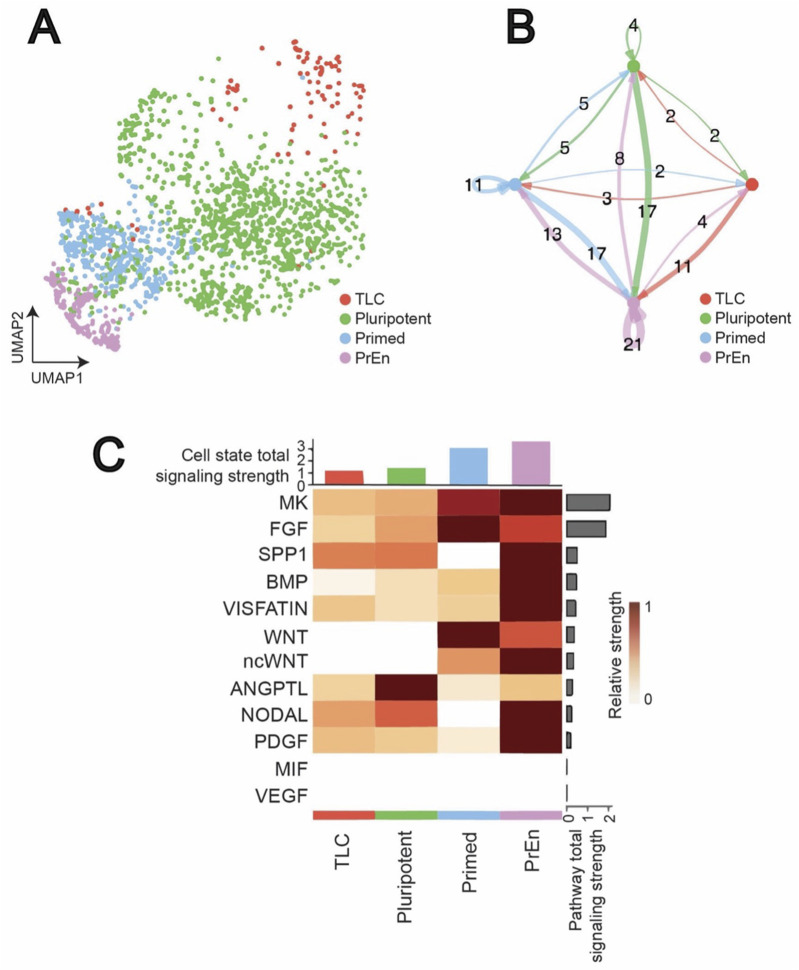
Intercellular communication landscape of ESCs in LB condition revealed by scRNA seq. **(A)** UMAP visualization of single-cell RNA-seq data of ESCs comparing the cellular landscape under the LB condition, highlighting major cell populations. **(B)** Circle plots depicting the number of inferred intercellular interactions among cell types in the LB condition as computed by CellChat. Edge thickness corresponds to interaction strength, while the arrow signifies the direction of the communication. **(C)** Overview of signalling pathway involvement in LB. The bar plot at the top shows the total signalling strength of a cell group by summarizing all signalling pathways displayed in the heatmap. The right grey bar plot shows the total signalling strength of a signalling pathway by summarizing all cell groups displayed in the heatmap.

Mouse ESCs are regulated by multiple cell signalling pathways (Huang et al., 2015). To understand the full extent of CCC mediated by signalling pathways, we first evaluated the previously reported scRNA-seq data of ESCs cultured in LB (Meharwade et al., 2023). As expected, based on distinct expression of marker genes we observed all 4 cell states—TLCs (*Gata2*, *Rell1, Amhr2)*, pluripotent (*Sox2, Nanog, Klf4*), primed (*Zic3*, *T, Fgf8*), and PrEn (*Gata6, Cdh2, Dab2*)—in ESCs cultured in LB (Meharwade et al., 2023) (Figure 2A; Supplementary Figure S1). This distribution indicates that LB supports broad cell state diversity and maintains cellular heterogeneity. Consistent with this, intercellular communication analysis using CellChat revealed a highly interconnected signalling network in the LB condition (Figure 2B). Each cell state received and transmitted paracrine signals, confirming extensive interactions across the system. The number of edges between nodes indicates that PrEn cells are major hubs of communication, receiving inputs from and sending outputs to all other states (e.g., a total of 30 signals between primed to PrEn, 15 signals between TLCs to PrEn, and 25 signals between pluripotent to PrEn). We further observed bidirectional communication between TLCs and all other cell states, suggesting that their maintenance relies on multiple regulatory interactions, with a preference for PrEn.

A closer examination of signalling patterns further highlighted the complexity of this communication. Estimating the total signalling strength of each cell state and heatmap visualization of top contributing pathways revealed that primed and PrEn cells contributed the majority of signalling output (Figure 2C). Pathways such as MK, FGF, SPP1 and BMP were some of the dominant drivers of communication, primarily originating from the primed and PrEn cells.

**FIGURE 3:**
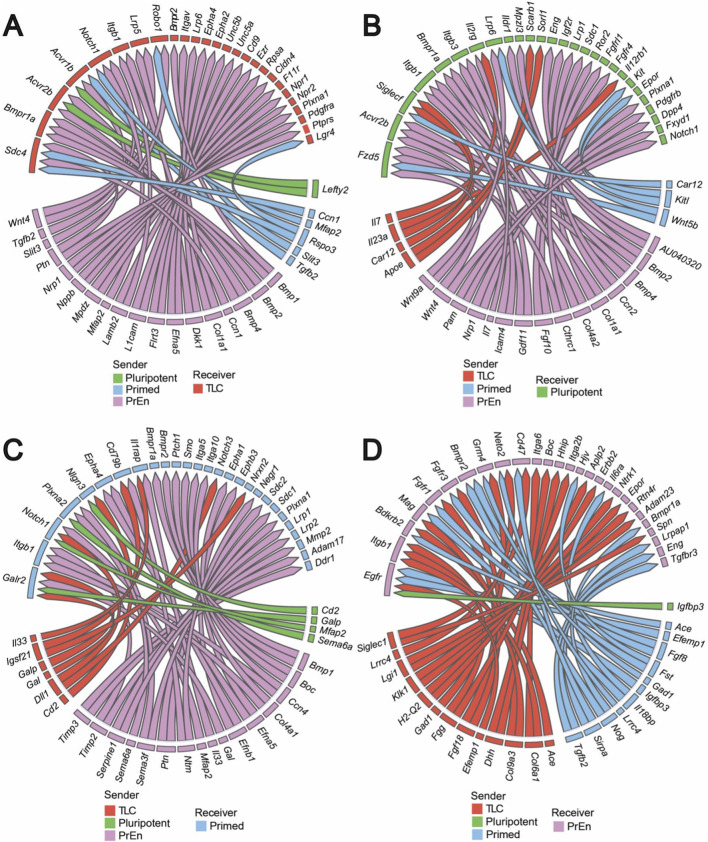
Differential NicheNet analysis reveals the top ligand-receptor pairs in the LB condition. Circos plots illustrating ligand–receptor interactions between all sender cell types and the **(A)** TLC, **(B)** pluripotent, **(C)** primed, and **(D)** PrEn receiver population in LB. Chords represent the strength and directionality of signalling inputs.

To specifically assess how TLCs are regulated within their environment, we performed receiver niche analysis for each cell state using NicheNet (Browaeys et al., 2020). NicheNet is a computational method that predicts how ligands from 1 cell type influence gene expression in another cell type. It integrates ligand–target regulatory networks with transcriptomic data to study cell–cell communication and its downstream effects. TLC-receiver analysis identified preferentially enriched ligands in pluripotent, primed and PrEn cells, and their corresponding receptors in TLCs (Figure 3A). Key ligand-receptor pairs included *Bmp4–Acvr2b*, *Wnt4–Lrp5*, *Tgfβ2–Acvr1b*, and *Lefty2–Acvr2b*, many of which belong to the FGF, WNT, BMP, and NODAL (TGF-β) families (Lafyatis, 2014; Xue et al., 2025; Ornitz and Itoh, 2015), which are crucial for stem cell maintenance, suggesting their role in regulating the TLC state (Meharwade et al., 2023; Xu and Liang, 2022; Chen et al., 2022). This data also verified that PrEn and primed cells provided the strongest input ligand signals to influence TLCs. In addition to these canonical pathways, we also observed adhesion-related ligands (e.g., *Ccn1*, *Flrt3*), suggesting that cell–cell contact cues may further modulate TLC plasticity (Karaulanov et al., 2006; Yang et al., 2018). Similar analysis for pluripotent, primed and PrEn as receiver cells revealed the extensive ligand-receptor pairs of FGF, BMP, NODAL (TGF-β), WNT, and PDGF pathways involved in regulating the stem cell states (Figures 3B–D).

Taken together, these data reveal that cell states within ESC populations communicate via an intricate network of paracrine signalling. Higher level of CCC correlates with high cell state heterogeneity in ESCs, suggesting a regulatory relationship. They also show that the extent of the TLC state is likely heavily influenced by paracrine signalling from pluripotent, primed, and PrEn cells.

### 3.2 Diminished CCC correlates with reduced heterogeneity

**FIGURE 4:**
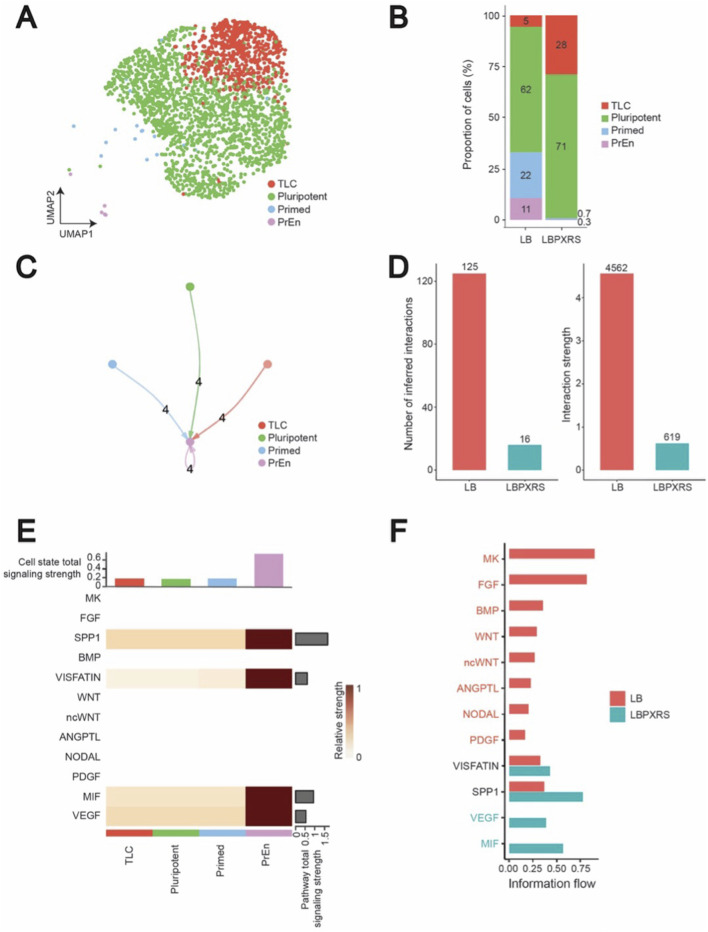
Comparative analysis of the intercellular communication landscape between LB and LBPXRS conditions. **(A)** UMAP visualization of the single-cell RNA-seq data of ESCs from the LBPXRS condition. **(B)** Barplot comparing the proportions of identified cell types in LB and LBPXRS. **(C)** Circle plots depicting the number of inferred intercellular interactions among cell types in the LBPXRS condition as computed by CellChat. Edge thickness corresponds to interaction strength, while the arrow signifies the direction of the communication. **(D)** Quantitative comparison of the total number of interactions and overall communication strength amongst LB and LBPXRS conditions. **(E)** Overview of signalling pathway involvement in LBPXRS. The bar plot on the top shows the total signalling strength of a cell group by summarizing all signalling pathways displayed in the heatmap. The right grey bar plot shows the total signalling strength of a signalling pathway by summarizing all cell groups displayed in the heatmap. **(F)** Stacked barplot of the information flow associated with each pathway across LB and LBPXRS conditions.

To validate whether the extent of signalling activity observed in ESC culture regulates cell state heterogeneity, we next evaluated scRNA-seq data of ESCs cultured in LBPXRS, together with LB conditions (Meharwade et al., 2023). In the LBPXRS condition, BMP-mediated cross-activation of the FGF, WNT, and NODAL (TGF-β) pathways is selectively inhibited using PD0325901, XAV939, and Resorcyclic lactone/SB431542, respectively (Meharwade et al., 2023). This condition has also been demonstrated to strikingly alter cell state composition by enriching TLCs and pluripotent cells and nearly eliminating primed and PrEn cells (Meharwade et al., 2023) (Figure 4A). Quantitative assessment of cell-type proportions confirmed these observations compared to LB, LBPXRS culture showed a ∼23% enrichment of TLCs and a ∼9% increase in pluripotent cells, accompanied by a drastic reduction of primed cells to 0.7% and PrEn to 0.3% of the population (Figure 4B). These findings indicate that reduced cellular heterogeneity in the LBPXRS condition narrows the cell identity landscape and diminishes cell signalling. Thus, with overall low signalling activity and altered cell state composition, scRNA-seq data from LBPXRS is highly suitable for evaluating the role of CCC.

Consistent with reduced heterogeneity, CellChat analysis of LBPXRS scRNA-seq data showed a simplified and sparsely connected signalling network, indicating that intercellular communication is significantly restricted (Figure 4C). Quantitative assessment of global communication metrics further supported this observation, with LB exhibiting substantially more interactions (125 vs. 16) and stronger overall interaction strength (4.562 vs. 0.619) compared to LBPXRS (Figure 4D). Thus, while LB fosters a dynamic and responsive niche environment that supports greater diversity of cell states, LBPXRS restricts signalling activity and promotes a more uniform cellular landscape.

To further dissect these differences, we performed pathway-level comparative analysis (Figure 4E). LB cultures engaged a broad array of signalling routes, including FGF, BMP, WNT, NODAL, and PDGF, whereas, as expected, these pathways were absent under LBPXRS (Figures 2C, 4E,F). Instead, exclusive communication in LBPXRS was limited to MIF and VEGF signalling, while common pathways between the two conditions included SPP1 and VISFATIN. Notably, interaction strength appeared inflated for the PrEn state due to its very low cell count, underscoring the importance of accounting for relative cell-type abundance in CCC inference. These findings highlight that the molecular signals supporting cell identity and intercellular communication are strongly shaped by culture conditions, with LB providing a signalling-rich environment that promotes plasticity and cell state transitions, whereas LBPXRS enforces a signalling-restricted space characterized by reduced cell state heterogeneity.

These data indicate that inhibition of FGF, WNT, and NODAL (TGF-β) pathways in LBPXRS culture constrains intercellular communication, leading to a simplified signalling network and restricted usage of pathways. This reduction in communication capacity underlies the observed loss of heterogeneity and emphasizes the critical role of culture-dependent signalling in shaping cellular identity landscapes.

### 3.3 BMP and NODAL (TGF-β) driven signalling regulates the TLCs

**FIGURE 5:**
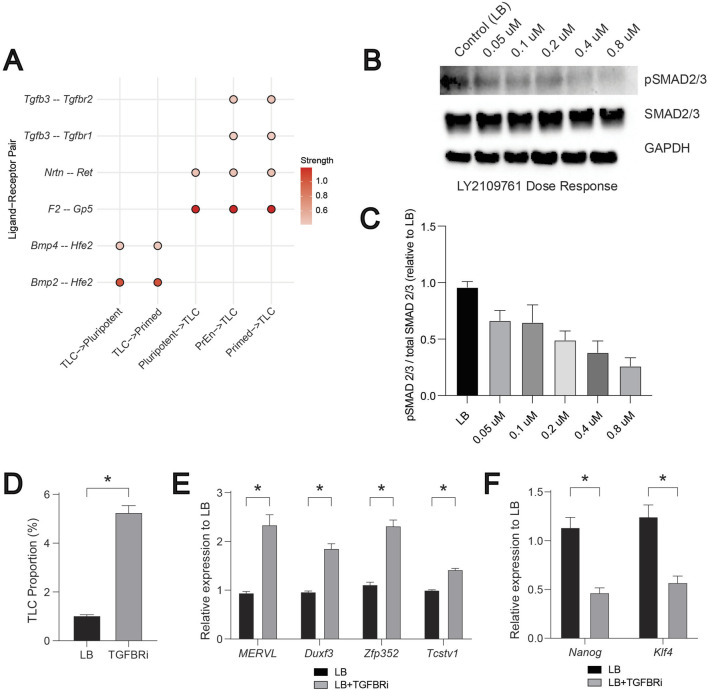
BMP and NODAL (TGF-β) driven signalling regulates the TLCs. **(A)** A compiled dot plot of differentially expressed ligand–receptor (LR) pairs across “TLC-senders” and “TLC-receivers” from FGF, BMP, WNT, NODAL (TGF-β), and PDGF signalling pathways. Scale represents the strength of the interaction potential. **(B)** Representative Western blot of NODAL (TGF-β) pathway activity in ESCs treated with increasing doses of the TGF-β receptor inhibitor LY2109761. Phosphorylated and total SMAD2/3 levels are shown, with GAPDH used as a loading control. **(C)** Quantification of pathway activity expressed as the ratio of phosphorylated to total SMAD2/3 signal relative to LB (n = 3). **(D)** Bar graph showing the proportion of TLCs (MERVL-tdTomato positive cells) in LB and TGFBRi conditions. **(E)** Relative mRNA levels of TLC genes: *MERVL, Duxf3, Zfp352,* and *Tcstv1* quantified by qRT-PCR, normalized to housekeeping gene (*Gapdh*), and fold change is relative to LB controls (n = 3). **(F)** Relative mRNA levels of pluripotent genes: *Klf4* and *Nanog* quantified by qRT-PCR, normalized to housekeeping gene (*Gapdh*), and fold change is relative to LB controls (n = 3). In Figures 5C–F, error bars represent the standard error of the mean. For Figures 5C–F,T-test is applied to show statistical significance.

Focusing on the LB data, we next examined ligand-receptor interactions using an interaction strength threshold of 0.4 to identify the exclusive and most confident communication routes involving TLCs. Based on the overlap between pathways identified by CellChat and Differential NicheNet, we focused on FGF, BMP, NODAL (TGF-β), WNT, and PDGF, as these have previously been shown to regulate ESCs (Meharwade et al., 2023). We did not consider other predominant pathways, such as the MK pathway observed in CellChat analysis (Figure 2C), as their ligand-receptor pairs were not significant in the differential NicheNet analysis. We observed limited ligand-receptor interactions, mediated by *Bmp2/4-Hfe2*, with TLCs as senders and pluripotent and primed cells serving as receivers (Figure 5A). On the contrary, we observed multiple ligand-receptor interactions, involving *F2-Gp5*, *Nrtn-Ret*, *Tgfb3-Tgfbr1* and *Tgfb3-Tgfbr2*, with pluripotent, PrEn, and primed cells as senders and TLCs as receivers (Figure 5A). This suggested a potentially unique incoming signalling axis to TLCs. These patterns suggest that TLCs act as both senders and receivers, engaging in multiple signalling systems to influence the states of their neighbouring cells.

We have previously demonstrated the crucial role of BMP2/4 for inducing the TLC state (Meharwade et al., 2023). Inhibition of BMP signalling using LDN193189, a selective inhibitor of BMP receptors (Cuny et al., 2008), in the LB condition drastically reduced the TLC proportions. In addition, increasing the dose of BMP correspondingly increased the proportion of TLCs and the expression of TLC genes (Meharwade et al., 2023). These experimental data further render validation of the above predicted role of BMP signalling in CCC, consistent with its predicted role in influencing the receiver cell states to promote the TLC state (Meharwade et al., 2023). To similarly validate the predicted role of NODAL (TGF-β) signalling, we next targeted the TGFBR1/2 (“receiver” pair) (Figure 5A). We chose LY2109761, a known specific small molecule inhibitor, to inhibit TGFBR1/2 activation (Melisi et al., 2008). We used ESCs harbouring MERVL-tdTomato live-cell fluorescent reporter to evaluate the changes in TLC proportions using flow cytometry (Meharwade et al., 2023; Macfarlan et al., 2012). First, by performing a dose-response analysis, we identified 0.8 μM LY2109761 as the concentration that maximally inhibits the activation of NODAL (TGF-β) signalling (Figures 5B,C). Next, we assessed the change in TLC proportion using flow cytometry and the expression of TLC genes by qPCR upon inhibition of TGF-β signalling with LY2109761. We observed a ∼5-fold increase in TLC proportion and ∼2-fold increase in the expression of multiple TLC genes (*MERVL*, *Duxf3*, *Zfp352*, *Tcstv1*) (Figures 5D,E). Similar measurements of pluripotent genes (*Klf4* and *Nanog)* showed a significant reduction in their expression upon treatment with LY2109761 (Figure 5F). These data demonstrate that TGF-β-mediated communication to the TLCs acts as a negative regulator of the TLC state. As a result, inhibition of TGF-β-mediated signalling increased the TLC state and decreased the pluripotent state, suggesting an overall effect on cell-state heterogeneity in the ESC system.

Together, these results demonstrate that TLC abundance is tightly regulated by the BMP and NODAL (TGF-β) signalling as positive and negative regulators, respectively. It also demonstrates that TLCs integrate multiple extrinsic growth factor signalling in order to either maintain or exit the TLC state.

### 3.4 In-silico perturbation of BMP-associated transcription factors reveals cell state transition dynamics

**FIGURE 6:**
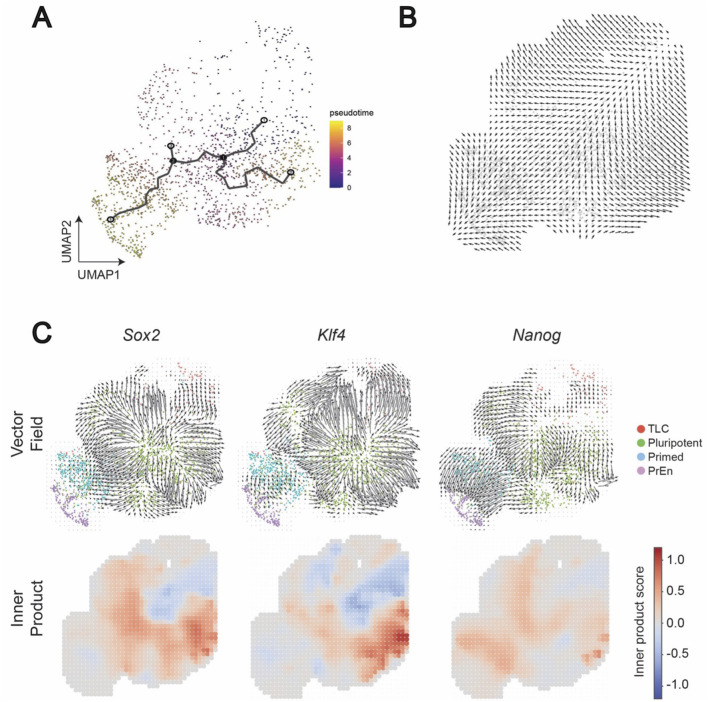
Developmental flow inference and cell state gene perturbation simulations in LB. **(A)** Monocle pseudotime inference. The TLC population was designated as the source state to reconstruct developmental trajectories. **(B)** CellOracle baseline developmental flow. CellOracle computed a pseudotime-like baseline vector field directly from the transcriptome manifold, visualized on a digitized grid. This developmental flow reflects the intrinsic transcriptional dynamics of system. **(C)** CellOracle transcription factor knockout simulations. For each pluripotent cell state transcription factor (*Sox2*, *Klf4*, *Nanog),* perturbation outcomes are shown in two subpanels: (top) vector fields illustrating local shifts in developmental trajectories compared to baseline flow, (bottom) inner product heatmaps quantifying alignment between perturbation vectors and baseline flow (positive values indicate reinforcement, negative values indicate opposition).

Cell signalling often orchestrates cell fate decisions via specific transcription factors (Di Giammartino et al., 2019). To determine how BMP signalling influences cell fate dynamics of TLCs and other cell states, we utilized CellOracle, which predicts cell fate dynamics by integrating gene regulatory network inference (Kamimoto et al., 2023). Since CellOracle requires a pseudotime variable as input to construct its developmental vector field, we first inferred pseudotime trajectories with Monocle (Trapnell et al., 2014), using TLCs as the source to anchor developmental time (Figure 6A). The predicted pseudotime trajectory closely matches the natural developmental cell fate transitions of preimplantation development (Meharwade et al., 2023; Du and Wu, 2024). To establish a dynamics-aware baseline that is unaffected by perturbations, we calculated CellOracle’s digitized developmental flow, a vector field derived directly from the transcriptomic landscape using the Monocle pseudotime variable as input (Figure 6B).

To verify that the CellOracle correctly predicts the stem cell state dynamic transitions, we first perturbed the well-known pluripotency TFs. We estimated the point of divergence for each gene of interest (the start of deviation from the developmental flow) and the inner product (positive = reinforcement of the developmental flow; negative = opposition against the developmental flow). The perturbation vector fields for pluripotent genes *Sox2*, *Klf4*, and *Nanog* (Di Giammartino et al., 2019; Avilion et al., 2003; Pan and Thomson, 2007) all diverge from within the pluripotent population (Figure 6C). *Sox2* and *Klf4* show negative inner products from the pluripotent population to the TLC population, and positive inner products converging in the primed state. *Nanog* generally exhibits weaker negative inner products, while positive inner products extend into more differentiated states. These results, by recapitulating the expected exit from pluripotency, indicate that CellOracle provides biologically coherent predictions of gene function during stem cell state transitions.

**FIGURE 7:**
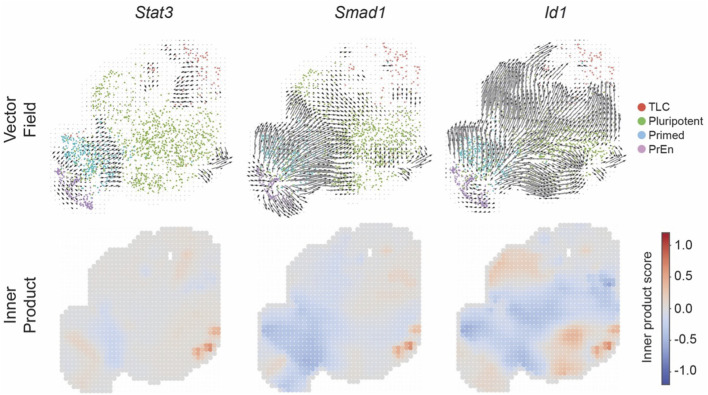
Signalling pathway target transcription factor perturbation simulations in LB. CellOracle transcription factor knockout simulations. For each transcription factor (*Stat3*, *Smad1* and *Id1*), perturbation outcomes are shown in two subpanels: (top) vector fields illustrating local shifts in developmental trajectories compared to baseline flow, (bottom) inner product heatmaps quantifying alignment between perturbation vectors and baseline flow (positive values indicate reinforcement, negative values indicate opposition).

Next, we tested the role of key TFs controlled by cell signalling pathways LIF and BMP. Through induction of Klf4 and Id gene expression, LIF and BMP signalling pathways support the self-renewal and pluripotent identity of ESCs, maintaining them in a pluripotent state (Niwa et al., 1998; van Oosten et al., 2012; Ying et al., 2003). Perturbation of the LIF pathway TF *Stat3* (Niwa et al., 1998) diverged the vector field in the primed state (Figure 7). Inner products are mostly negative in the primed state, with positive alignment in TLC and PrEn, indicating a modest, context-dependent modulation. In contrast, perturbation of BMP pathways TFs, *Smad1* and *Id1*, had a major impact on the vector fields (Ying et al., 2003; Trompouki et al., 2011). *Smad1* perturbation diverged the vector field in PrEn and showed predominantly negative inner products that converged toward the pluripotent state. *Id1*, another BMP pathway target, diverged in primed and produced strong, extensive negative inner products across primed and pluripotent regions, converging toward the TLC state.

Together, these data further confirm the crucial role of BMP signalling in regulating the TLC state as well as other stem cell states of early embryonic development (Meharwade et al., 2023; Graham et al., 2014). They provide a mechanistic model to link normal developmental flow with variable transcriptional responses induced by cell signalling.

## 4 Discussion

Our findings highlight how paracrine signalling influences ESC cell fate dynamics, with TLCs continually shaped by signals from their pluripotent, primed, and PrEn neighbours. Such intercellular communication plays a crucial role in early embryonic development. For instance, in the preimplantation embryo, FGF4 secreted by epiblast precursors instructs adjacent inner cell mass cells to adopt a PrEn identity, exemplifying a paracrine-driven lineage specification (Kang et al., 2017). At the peri-implantation stage, reciprocal signalling between tissues orchestrates morphogenetic remodelling–epiblast-derived factors help sustain extraembryonic lineages, such as trophectoderm and endoderm (Vr et al., 2022), and emerging morphogen gradients break symmetry to establish body axes (Cheng et al., 2025). Notably, localized BMP4 signals trigger the WNT and NODAL pathways across the epiblast and visceral endoderm, initiating the formation of the anterior-posterior axis (Cheng et al., 2025).

While our previous work employed ssGSEA-based pathway enrichment analysis to assess BMP and NODAL signalling activity (Meharwade et al., 2023), this approach was limited in its ability to determine whether these pathways facilitate intercellular communication or influence neighbouring cell populations. In the present study, we extend beyond pathway-level enrichment by interrogating BMP and NODAL signalling through a ligand–receptor interaction framework, thereby enabling the identification of putative sender–receiver relationships among TLCs.

Such parallels suggest that spatially confined microenvironments act as information-processing hubs: cells integrate multiple concurrent morphogen inputs, and developmental systems filter out stochastic noise to ensure robust patterning (Davies and Albeck, 2018). In essence, the embryo’s signalling niches dynamically balance stem cell self-renewal while maintaining developmental equilibrium. This perspective also implies that by manipulating paracrine signalling, we can steer cell plasticity in culture. Indeed, experimentally tuning key pathways (e.g., inhibiting FGF, WNT, and NODAL while enhancing BMP signals) biases ESC populations toward the TLC state (Meharwade et al., 2023). Harnessing these principles could enable engineered control of cell fate *in vitro*, allowing us to recapitulate specific *in vivo* cell fate decision-making of embryonic development.

In our study, we observed that a more diverse intercellular communication network is associated with increased cellular heterogeneity. This likely reflects the contribution of distinct transcriptional profiles across individual cells, which together enhance the stability and resilience of the signalling landscape. For example, in skin and oesophageal epithelia, tissue homeostasis is maintained by stem cells that balance self-renewal and differentiation during proliferation. This balance is tightly regulated, and its disruption through intrinsic or intercellular signalling or cellular communication defects can lead to pathological states such as cancer (Valls and Esposito, 2022). Another example is of tumour heterogeneity driven by the extensive network of interactions within the tumour microenvironment. Diverse signalling cues exchanged between neighbouring cells shape the transcriptional programs of receiver cells, giving rise to distinct cellular phenotypes and behaviours such as tumorigenesis, altered immune infiltration, and increased therapy resistance (Chen et al., 2023). Together, these examples support the idea that broader signalling diversity directly contributes to the emergence and maintenance of heterogeneous cellular states. Previous work has highlighted the role of paracrine and autocrine signalling in regulating pluripotency and lineage commitment (Przybyla and Voldman, 2012; Dalton, 2013), and our findings extend this by suggesting that heterogeneity itself may serve as a stabilizing factor in maintaining pluripotent states.

The importance of the principle that heterogeneity influences the signalling environment becomes evident in disease contexts such as intrahepatic cholangiocarcinoma (ICC), where reduced cellular diversity coincides with a less intricate outgoing signalling network (Zhou et al., 2023). Interestingly, the cancer cell populations exhibited greater heterogeneity and stronger outgoing signalling compared to their normal counterparts, suggesting that heterogeneity may confer adaptive advantages in pathological settings. Collectively, these results support the notion that cellular heterogeneity functions as a general mechanism for population stability and adaptability. This has important implications not only for understanding tumour progression but also for optimizing stem cell culture conditions and developing therapeutic strategies that aim to preserve or manipulate cell states by modulating intercellular communication.

Our findings reveal distinct effects of NODAL (TGF-β) pathway inhibition on TLC regulation, underscoring their differential contributions to cell state control. Inhibition of TGF-β signalling produced both a marked increase in TLC frequency and an upregulation of TLC gene expression, indicating that suppression of this pathway promotes the expansion or stabilization of the TLC population. These findings suggest that TGF-β signalling negatively regulates both the transcriptional and cellular maintenance of TLCs. Together, the data highlight a potential inhibitory role of TGF-β signalling in TLC maintenance.

The *in silico* gene perturbation behaviours we observed between cell state genes and pathway targets can be explained by their distinct roles in regulation. Perturbations of pluripotent cell state markers were first performed to validate that the model correctly reproduces expected transcriptional behaviours within the pluripotent population, ensuring that predicted cell fate shifts reflect biologically coherent responses. Cell state genes, such as *Sox2*, *Klf4*, and *Nanog*, primarily serve as indicators of pluripotent identity. As expected, their perturbation triggers a pluripotency exit, resulting in positive inner products that align with differentiation. This finding also aligns with previous research that suggests *Nanog* is not strictly essential for self-renewal, as altering its levels can influence lineage commitment (Chambers and Tomlinson, 2009), and that *Sox2* and *Klf4* primarily determine pluripotent identity (Takahashi and Yamanaka, 2006). We then extended the perturbations to pathway TFs like *Stat3*, *Smad1*, and *Id1*, which actively help stabilize or redirect cell fate decisions. For instance, *Stat3* activation downstream of LIF is both necessary and sufficient to maintain self-renewal (Niwa et al., 1998; Matsuda et al., 1999), and BMP signalling maintains pluripotency through *Smad1* and *Id1*-mediated suppression of stem cell differentiation (Ying et al., 2003). These results imply that perturbing specific signalling pathway TFs dynamically alter the natural developmental cell fate transitions.

## 5 Perspectives and limitations

While this study provides insights into intercellular communication in ESCs, several limitations should be considered. First, our analysis does not address the effects of autocrine signalling on the TLC state. Autocrine loops are known to influence cell fate decisions (Przybyla and Voldman, 2012), and incorporating this dimension would refine our understanding of how individual cells integrate self-derived vs. extrinsic cues. Second, all our validations were performed in a two-dimensional *in vitro* setting. Although 2D cultures are tractable and widely used, they lack the spatial architecture and mechanical context of early embryonic environments. Extending these approaches to three-dimensional models such as embryoid bodies or organoid systems would provide a more physiologically relevant framework and enhance the translational applicability of our findings. Our study is based on mouse ESCs, in which cell-state heterogeneity and the TLC state are well characterized. Because early developmental stages and signalling pathways are broadly conserved, the CCC mechanisms we describe here may also be relevant to human ESCs, which have been shown to exhibit both marked differences and similarities (Gabdoulline et al., 2015).

## Data Availability

Publicly available datasets were analyzed in this study. This data can be found here: https://www.ncbi.nlm.nih.gov/geo/query/acc.cgi?acc=GSE198384. Gene Expression Omnibus repository, Accession number: GSE198384.

## References

[B1] Artus J. Piliszek A. Hadjantonakis A. K. (2011). The primitive endoderm lineage of the mouse blastocyst: sequential transcription factor activation and regulation of differentiation by Sox17. Dev. Biol. 350 (2), 393–404. 10.1016/j.ydbio.2010.12.007 21146513 PMC3461954

[B2] Avilion A. A. Nicolis S. K. Pevny L. H. Perez L. Vivian N. Lovell-Badge R. (2003). Multipotent cell lineages in early mouse development depend on SOX2 function. Genes Dev. 17 (1), 126–140. 10.1101/gad.224503 12514105 PMC195970

[B3] Browaeys R. Saelens W. Saeys Y. (2020). NicheNet: modeling intercellular communication by linking ligands to target genes. Nat. Methods 17 (2), 159–162. 10.1038/s41592-019-0667-5 31819264

[B4] Buecker C. Srinivasan R. Wu Z. Calo E. Acampora D. Faial T. (2014). Reorganization of enhancer patterns in transition from naive to primed pluripotency. Cell Stem Cell 14 (6), 838–853. 10.1016/j.stem.2014.04.003 24905168 PMC4491504

[B5] Casey M. J. Stumpf P. S. MacArthur B. D. (2020). Theory of cell fate. Wiley Interdiscip. Rev. Syst. Biol. Med. 12 (2), e1471. 10.1002/wsbm.1471 31828979 PMC7027507

[B6] Chambers I. Tomlinson S. R. (2009). The transcriptional foundation of pluripotency. Development 136 (14), 2311–2322. 10.1242/dev.024398 19542351 PMC2729344

[B7] Chehelgerdi M. Behdarvand Dehkordi F. Chehelgerdi M. Kabiri H. Salehian-Dehkordi H. Abdolvand M. (2023). Exploring the promising potential of induced pluripotent stem cells in cancer research and therapy. Mol. Cancer 22 (1), 189. 10.1186/s12943-023-01873-0 38017433 PMC10683363

[B9] Chen G. Yin S. Zeng H. Li H. Wan X. (2022). Regulation of embryonic stem cell self-renewal. Life (Basel) 12 (8), 1151. 10.3390/life12081151 36013330 PMC9410528

[B10] Chen L. X. Zeng S. J. Liu X. D. Tang H. B. Wang J. W. Jiang Q. (2023). Cell-cell communications shape tumor microenvironment and predict clinical outcomes in clear cell renal carcinoma. J. Transl. Med. 21 (1), 113. 10.1186/s12967-022-03858-x 36765369 PMC9921120

[B11] Cheng D. Clark C. T. Smith Q. (2025). Advances in engineered models of peri-gastrulation. iScience 28 (6), 112659. 10.1016/j.isci.2025.112659 40510116 PMC12159512

[B12] Choi Y. J. Lin C. P. Risso D. Chen S. Kim T. A. Tan M. H. (2017). Deficiency of microRNA miR-34a expands cell fate potential in pluripotent stem cells. Science 355 (6325), eaag1927. 10.1126/science.aag1927 28082412 PMC6138252

[B13] Cooper G. M. (2000). The cell: a molecular approach. 2nd Edition ed2000.

[B14] Cuny G. D. Yu P. B. Laha J. K. Xing X. Liu J.-F. Lai C. S. (2008). Structure–activity relationship study of bone morphogenetic protein (BMP) signaling inhibitors. Bioorg. & Med. Chem. Lett. 18 (15), 4388–4392. 10.1016/j.bmcl.2008.06.052 18621530 PMC2570262

[B15] Dalton S. (2013). Signaling networks in human pluripotent stem cells. Curr. Opin. Cell Biol. 25 (2), 241–246. 10.1016/j.ceb.2012.09.005 23092754 PMC3570582

[B16] Davies A. E. Albeck J. G. (2018). Microenvironmental signals and biochemical information processing: cooperative determinants of intratumoral plasticity and heterogeneity. Front. Cell Dev. Biol. 6, 44. 10.3389/fcell.2018.00044 29732370 PMC5921997

[B17] Di Giammartino D. C. Kloetgen A. Polyzos A. Liu Y. Kim D. Murphy D. (2019). KLF4 is involved in the organization and regulation of pluripotency-associated three-dimensional enhancer networks. Nat. Cell Biol. 21 (10), 1179–1190. 10.1038/s41556-019-0390-6 31548608 PMC7339746

[B18] Du P. Wu J. (2024). Hallmarks of totipotent and pluripotent stem cell states. Cell Stem Cell 31 (3), 312–333. 10.1016/j.stem.2024.01.009 38382531 PMC10939785

[B19] Gabdoulline R. Kaisers W. Gaspar A. Meganathan K. Doss M. X. Jagtap S. (2015). Differences in the early development of human and Mouse embryonic stem cells. PLoS One 10 (10), e0140803. 10.1371/journal.pone.0140803 26473594 PMC4608779

[B20] Graham S. J. L. Wicher K. B. Jedrusik A. Guo G. Herath W. Robson P. (2014). BMP signalling regulates the pre-implantation development of extra-embryonic cell lineages in the mouse embryo. Nat. Commun. 5 (1), 5667. 10.1038/ncomms6667 25514175 PMC4338527

[B21] Haghverdi L. Ludwig L. S. (2023). Single-cell multi-omics and lineage tracing to dissect cell fate decision-making. Stem Cell Rep. 18 (1), 13–25. 10.1016/j.stemcr.2022.12.003 36630900 PMC9860164

[B22] Huang G. Ye S. Zhou X. Liu D. Ying Q. L. (2015). Molecular basis of embryonic stem cell self-renewal: from signaling pathways to pluripotency network. Cell Mol. Life Sci. 72 (9), 1741–1757. 10.1007/s00018-015-1833-2 25595304 PMC4809369

[B23] Jin S. Guerrero-Juarez C. F. Zhang L. Chang I. Ramos R. Kuan C. H. (2021). Inference and analysis of cell-cell communication using CellChat. Nat. Commun. 12 (1), 1088. 10.1038/s41467-021-21246-9 33597522 PMC7889871

[B24] Jovic D. Liang X. Zeng H. Lin L. Xu F. Luo Y. (2022). Single-cell RNA sequencing technologies and applications: a brief overview. Clin. Transl. Med. 12 (3), e694. 10.1002/ctm2.694 35352511 PMC8964935

[B25] Kamimoto K. Stringa B. Hoffmann C. M. Jindal K. Solnica-Krezel L. Morris S. A. (2023). Dissecting cell identity via network inference and *in silico* gene perturbation. Nature 614 (7949), 742–751. 10.1038/s41586-022-05688-9 36755098 PMC9946838

[B26] Kang M. Garg V. Hadjantonakis A. K. (2017). Lineage establishment and progression within the inner cell mass of the mouse blastocyst requires FGFR1 and FGFR2. Dev. Cell 41 (5), 496–510.e5. 10.1016/j.devcel.2017.05.003 28552559 PMC5530874

[B27] Karaulanov E. E. Böttcher R. T. Niehrs C. (2006). A role for fibronectin-leucine-rich transmembrane cell-surface proteins in homotypic cell adhesion. EMBO Rep. 7 (3), 283–290. 10.1038/sj.embor.7400614 16440004 PMC1456895

[B28] Lafyatis R. (2014). Transforming growth factor β—at the centre of systemic sclerosis. Nat. Rev. Rheumatol. 10 (12), 706–719. 10.1038/nrrheum.2014.137 25136781

[B29] Lanner F. Rossant J. (2010). The role of FGF/Erk signaling in pluripotent cells. Development 137 (20), 3351–3360. 10.1242/dev.050146 20876656

[B30] Lee J. Kim N. Cho K. H. (2024). Decoding the principle of cell-fate determination for its reverse control. NPJ Syst. Biol. Appl. 10 (1), 47. 10.1038/s41540-024-00372-2 38710700 PMC11074314

[B31] Liang G. Zhang Y. (2013). Embryonic stem cell and induced pluripotent stem cell: an epigenetic perspective. Cell Res. 23 (1), 49–69. 10.1038/cr.2012.175 23247625 PMC3541668

[B32] Lu F. Zhang Y. (2015). Cell totipotency: molecular features, induction, and maintenance. Natl. Sci. Rev. 2 (2), 217–225. 10.1093/nsr/nwv009 26114010 PMC4477869

[B33] Meharwade T. Joumier L. Parisotto M. Huynh V. Lummertz da Rocha E. Malleshaiah M. (2023). Cross-activation of FGF, NODAL, and WNT pathways constrains BMP-signaling-mediated induction of the totipotent state in mouse embryonic stem cells. Cell Rep. 42 (5), 112438. 10.1016/j.celrep.2023.112438 37126449

[B34] Macfarlan T. S. Gifford W. D. Driscoll S. Lettieri K. Rowe H. M. Bonanomi D. (2012). Embryonic stem cell potency fluctuates with endogenous retrovirus activity. Nature 487 (7405), 57–63. 10.1038/nature11244 22722858 PMC3395470

[B35] Malleshaiah M. Padi M. Rue P. Quackenbush J. Martinez-Arias A. Gunawardena J. (2016). Nac1 coordinates a sub-network of pluripotency factors to regulate embryonic stem cell differentiation. Cell Rep. 14 (5), 1181–1194. 10.1016/j.celrep.2015.12.101 26832399 PMC4749452

[B36] Matsuda T. Nakamura T. Nakao K. Arai T. Katsuki M. Heike T. (1999). STAT3 activation is sufficient to maintain an undifferentiated state of mouse embryonic stem cells. EMBO J. 18 (15), 4261–4269. 10.1093/emboj/18.15.4261 10428964 PMC1171502

[B37] Melisi D. Ishiyama S. Sclabas G. M. Fleming J. B. Xia Q. Tortora G. (2008). LY2109761, a novel transforming growth factor beta receptor type I and type II dual inhibitor, as a therapeutic approach to suppressing pancreatic cancer metastasis. Mol. Cancer Ther. 7 (4), 829–840. 10.1158/1535-7163.MCT-07-0337 18413796 PMC3088432

[B38] Morrison S. J. Spradling A. C. (2008). Stem cells and niches: mechanisms that promote stem cell maintenance throughout life. Cell 132 (4), 598–611. 10.1016/j.cell.2008.01.038 18295578 PMC4505728

[B39] Movasat H. Giacopino E. Shahdoost A. Dorri Nokoorani Y. Abrbekouh A. H. Tahamtani Y. (2025). A systems view of cellular heterogeneity: unlocking the wheel of fate. Cell Syst. 16 (6), 101300. 10.1016/j.cels.2025.101300 40472847

[B40] Nichols J. Smith A. (2009). Naive and primed pluripotent states. Cell Stem Cell 4 (6), 487–492. 10.1016/j.stem.2009.05.015 19497275

[B55] NRC (US), Research IoMUCotBaBAoSC (2002). Stem cells and the future of regenerative medicine. Washington, D.C: National Academy Press.

[B41] Niwa H. Burdon T. Chambers I. Smith A. (1998). Self-renewal of pluripotent embryonic stem cells is mediated *via* activation of STAT3. Genes Dev. 12 (13), 2048–2060. 10.1101/gad.12.13.2048 9649508 PMC316954

[B42] Ornitz D. M. Itoh N. (2015). The fibroblast growth factor signaling pathway. Wiley Interdiscip. Rev. Dev. Biol. 4 (3), 215–266. 10.1002/wdev.176 25772309 PMC4393358

[B43] Pan G. Thomson J. A. (2007). Nanog and transcriptional networks in embryonic stem cell pluripotency. Cell Res. 17 (1), 42–49. 10.1038/sj.cr.7310125 17211451

[B44] Przybyla L. Voldman J. (2012). Probing embryonic stem cell autocrine and paracrine signaling using microfluidics. Annu. Rev. Anal. Chem. Palo Alto Calif. 5, 293–315. 10.1146/annurev-anchem-062011-143122 22524217 PMC4030416

[B45] Romito A. Cobellis G. (2016). Pluripotent stem cells: current understanding and future directions. Stem Cells Int. 2016, 9451492. 10.1155/2016/9451492 26798367 PMC4699068

[B46] Schrode N. Saiz N. Di Talia S. Hadjantonakis A. K. (2014). GATA6 levels modulate primitive endoderm cell fate choice and timing in the mouse blastocyst. Dev. Cell 29 (4), 454–467. 10.1016/j.devcel.2014.04.011 24835466 PMC4103658

[B47] Silva J. Smith A. (2008). Capturing pluripotency. Cell 132 (4), 532–536. 10.1016/j.cell.2008.02.006 18295569 PMC2427053

[B48] Singer S. J. (1992). Intercellular communication and cell-cell adhesion. Science 255 (5052), 1671–1677. 10.1126/science.1313187 1313187

[B49] Singer Z. S. Yong J. Tischler J. Hackett J. A. Altinok A. Surani M. A. (2014). Dynamic heterogeneity and DNA methylation in embryonic stem cells. Mol. Cell 55 (2), 319–331. 10.1016/j.molcel.2014.06.029 25038413 PMC4104113

[B50] Stuart T. Butler A. Hoffman P. Hafemeister C. Papalexi E. Mauck W. M. 3rd (2019). Comprehensive integration of single-cell data. Cell 177 (7), 1888–902 e21. 10.1016/j.cell.2019.05.031 31178118 PMC6687398

[B51] Su J. Song Y. Zhu Z. Huang X. Fan J. Qiao J. (2024). Cell-cell communication: new insights and clinical implications. Signal Transduct. Target Ther. 9 (1), 196. 10.1038/s41392-024-01888-z 39107318 PMC11382761

[B52] Takahashi K. Yamanaka S. (2006). Induction of pluripotent stem cells from mouse embryonic and adult fibroblast cultures by defined factors. Cell 126 (4), 663–676. 10.1016/j.cell.2006.07.024 16904174

[B53] Trapnell C. Cacchiarelli D. Grimsby J. Pokharel P. Li S. Morse M. (2014). The dynamics and regulators of cell fate decisions are revealed by pseudotemporal ordering of single cells. Nat. Biotechnol. 32 (4), 381–386. 10.1038/nbt.2859 24658644 PMC4122333

[B54] Trompouki E. Bowman T. V. Lawton L. N. Fan Zi P. Wu D.-C. DiBiase A. (2011). Lineage regulators direct BMP and wnt pathways to cell-specific programs during differentiation and regeneration. Cell 147 (3), 577–589. 10.1016/j.cell.2011.09.044 22036566 PMC3219441

[B56] Valls P. O. Esposito A. (2022). Signalling dynamics, cell decisions, and homeostatic control in health and disease. Curr. Opin. Cell Biol. 75, 102066. 10.1016/j.ceb.2022.01.011 35245783 PMC9097822

[B57] van Oosten A. L. Costa Y. Smith A. Silva J. C. (2012). JAK/STAT3 signalling is sufficient and dominant over antagonistic cues for the establishment of naive pluripotency. Nat. Commun. 3, 817. 10.1038/ncomms1822 22569365 PMC3567838

[B58] Vrij E. J. Scholte Op Reimer Y. S. Roa Fuentes L. Misteli Guerreiro I. Holzmann V. (2022). A pendulum of induction between the epiblast and extra-embryonic endoderm supports post-implantation progression. Development 149 (20), dev192310. 10.1242/dev.192310 35993866 PMC9534490

[B59] Wu S. Schmitz U. (2023). Single-cell and long-read sequencing to enhance modelling of splicing and cell-fate determination. Comput. Struct. Biotechnol. J. 21, 2373–2380. 10.1016/j.csbj.2023.03.023 37066125 PMC10091034

[B60] Wu G. Liang Y. Xi Q. Zuo Y. (2025). New insights and implications of cell-cell interactions in developmental biology. Int. J. Mol. Sci. 26 (9), 3997. 10.3390/ijms26093997 40362237 PMC12072105

[B61] Xu H. Liang H. (2022). The regulation of totipotency transcription: perspective from *in vitro* and *in vivo* totipotency. Front. Cell Dev. Biol. 10, 1024093. 10.3389/fcell.2022.1024093 36393839 PMC9643643

[B62] Xue C. Chu Q. Shi Q. Zeng Y. Lu J. Li L. (2025). Wnt signaling pathways in biology and disease: mechanisms and therapeutic advances. Signal Transduct. Target. Ther. 10 (1), 106. 10.1038/s41392-025-02142-w 40180907 PMC11968978

[B63] Yang R. Chen Y. Chen D. (2018). Biological functions and role of CCN1/Cyr61 in embryogenesis and tumorigenesis in the female reproductive system. Mol. Med. Rep. 17 (1), 3–10. (Review). 10.3892/mmr.2017.7880 29115499 PMC5780141

[B64] Yang S. H. Andrabi M. Biss R. Murtuza Baker S. Iqbal M. Sharrocks A. D. (2019). ZIC3 controls the transition from naive to primed pluripotency. Cell Rep. 27 (11). 10.1016/j.celrep.2019.05.026 31189106 PMC6581693

[B65] Yang S. Cho Y. Jang J. (2021). Single cell heterogeneity in human pluripotent stem cells. BMB Rep. 54 (10), 505–515. 10.5483/BMBRep.2021.54.10.094 34488931 PMC8560460

[B66] Ying Q. L. Nichols J. Chambers I. Smith A. (2003). BMP induction of Id proteins suppresses differentiation and sustains embryonic stem cell self-renewal in collaboration with STAT3. Cell 115 (3), 281–292. 10.1016/s0092-8674(03)00847-x 14636556

[B67] Zhou Z. Q. Zhang Y. Xu Z. Y. Tang X. L. Chen X. H. Guan J. (2023). Dissecting cellular heterogeneity and intercellular communication in cholangiocarcinoma: implications for individualized therapeutic strategies. Front. Genet. 14, 1241834. 10.3389/fgene.2023.1241834 38239853 PMC10794609

