# Supplementary figures and images for: BMP and NODAL paracrine signalling regulate the totipotent-like cell state in embryonic stem cells
Source: Front Cell Dev Biol. 2026 Jan 23;13:1720355. doi: 10.3389/fcell.2025.1720355 (PMC12876259; doi:10.3389/fcell.2025.1720355)

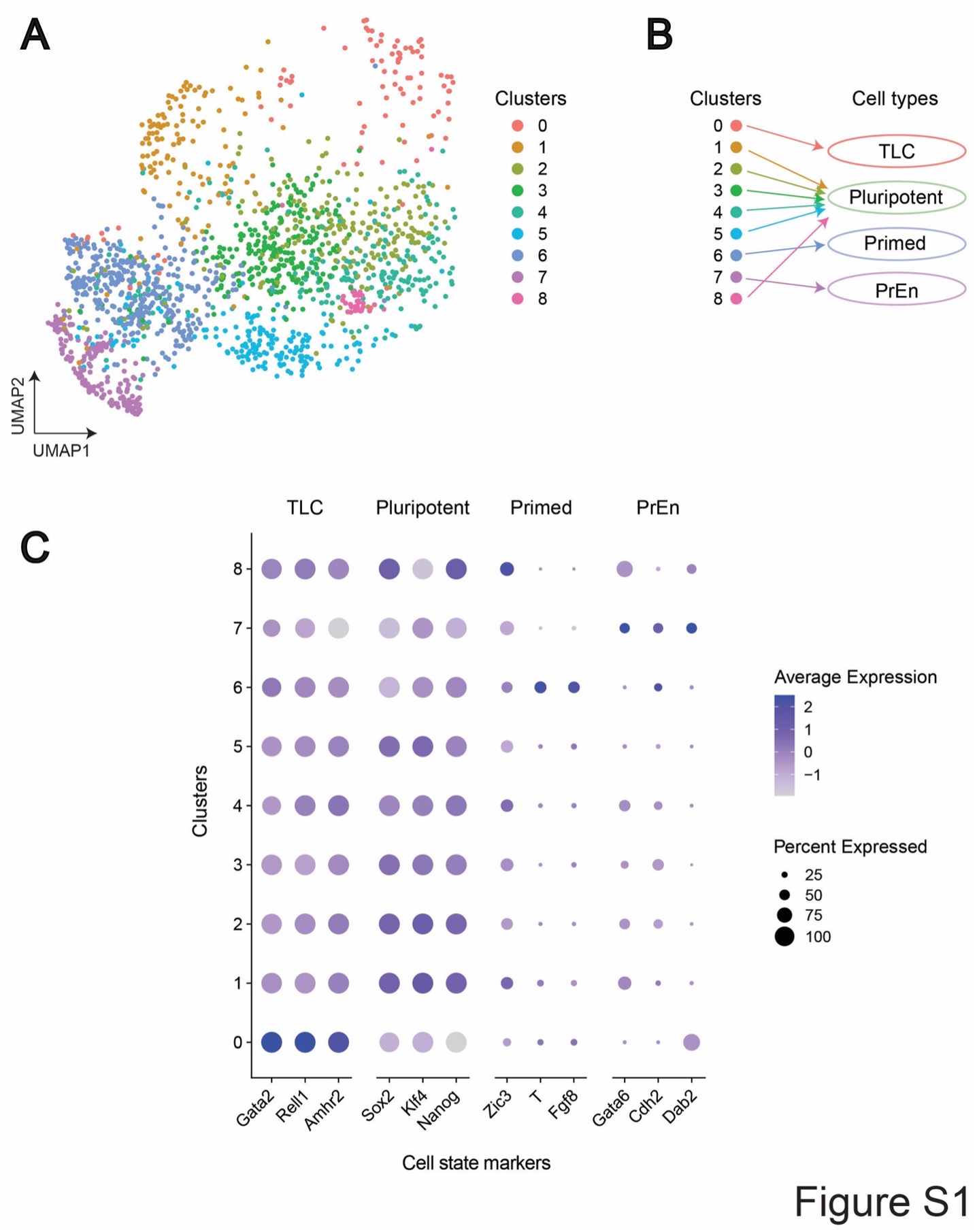

Supplement: Supplementary file 1 [file Image1.jpeg]
